# Enhancing the energy density of safer Li-ion batteries by combining high-voltage lithium cobalt fluorophosphate cathodes and nanostructured titania anodes

**DOI:** 10.1038/srep20656

**Published:** 2016-02-16

**Authors:** Gregorio F. Ortiz, María C. López, Yixiao Li, Matthew J. McDonald, Marta Cabello, José L. Tirado, Yong Yang

**Affiliations:** 1 Inorganic Chemistry Laboratory, University of Córdoba, Marie Curie Building, Campus of Rabanales, E-14071 Córdoba, Spain; 2 State Key Laboratory of Physical Chemistry of Solid Surfaces, Department of Chemistry, College of Chemistry and Chemical Engineering, Xiamen University, Xiamen 361005, P. R. China

## Abstract

Recently, Li-ion batteries have been heavily scrutinized because of the apparent incompatibility between safety and high energy density. This work report a high voltage full battery made with TiO_2_/Li_3_PO_4_/Li_2_CoPO_4_F. The Li_2_CoPO_4_F cathode and TiO_2_ anode materials are synthesized by a sol–gel and anodization methods, respectively. X-ray diffraction (XRD) analysis confirmed that Li_2_CoPO_4_F is well-crystallized in orthorhombic crystal structure with *Pnma* space group. The Li_3_PO_4_-coated anode was successfully deposited as shown by the (011) lattice fringes of anatase TiO_2_ and (200) of γ-Li_3_PO_4_, as detected by HRTEM. The charge profile of Li_2_CoPO_4_F versus lithium shows a plateau at 5.0 V, revealing its importance as potentially high-voltage cathode and could perfectly fit with the plateau of anatase anode (1.8–1.9 V). The full cell made with TiO_2_/Li_3_PO_4_/Li_2_CoPO_4_F delivered an initial reversible capacity of 150 mA h g^−1^ at C rate with good cyclic performance at an average potential of 3.1–3.2 V. Thus, the full cell provides an energy density of 472 W h kg^−1^. This full battery behaves better than TiO_2_/Li_2_CoPO_4_F. The introduction of Li_3_PO_4_ as buffer layer is expected to help the cyclability of the electrodes as it allows a rapid Li-ion transport.

Li-ion technology is now mature enough to meet the exacting demands of portable electronic devices and even electric vehicles. However, recently Li-ion batteries (LIBs) have come under heavy scrutiny because of an apparent incompatibility between safety and high energy density. The capabilities of LIBs are governed by the chemistry of the cathode, which almost exclusively utilizes transition metal insertion/intercalation reactions. The cathode material is not only the most expensive part of the battery but also the primary limitation on the electrochemical performance. It is thus desirable to find high voltage cathode materials with high capacity (xLi ≫ 1) and good electrolyte stability. LiNi_0.5_Mn_1.5_O_4_ spinel, LiCoPO_4_ olivine, LiNiVO_4_ inverse spinel and Li_2_CoPO_4_F fluorophosphates are currently considered to be the most promising 5-V cathode voltage materials available^1^,^2^,^3^,^4^,^5^,^6^. On the other side of the battery, there are a large number of possibilities for anodes to be combined with cathodes, but some of the most outstanding anodes with respect to safety performance are Li_4_Ti_5_O_12_ and TiO_2_, which can replace carbonaceous materials^7^,^8^,^9^,^10^. The TiO_2_ electrodes vs. Li_2_CoPO_4_F can be considered safer than other Li-ion systems based on carbon anodes, due to the higher working voltage of the anode that avoids lithium electrodeposition, which is well known to jeopardize safety, while energy density is preserved or even improved by the use of the high-voltage cathode.

Up until now, one of the more impressive LIB electrochemical performances has been seen in the Li_4_Ti_5_O_12_/Li_2_CoPO_4_F, primarily because it exhibits a voltage plateau at about 3.4 V which is higher than that of a Li_4_Ti_5_O_12_/LiFePO_4_ full cell at ~1.9 V^11^,^12^. However, an unresolved problem with the former system is that capacity decays abruptly in the first few cycles, and as of yet no improvement in cycling performance has been achieved. As far as we know, there are no reports in the literature dealing with TiO_2_/Li_2_CoPO_4_F that can reach theoretical energy densities above 450 W h kg^**−**1^, performance close to the demands of modern applications.

In order to improve the cyclability of high voltage LIBs, the effects of a surface treatment of lithium phosphate on a full cell made with Li_2_CoPO_4_F as cathode and TiO_2_ as anode were studied. This report shows how the electrochemical performance of this material compared very favourably with Li_3_PO_4_-free electrodes. The introduction of an inactive matrix such as Li_3_PO_4_ for use as a buffer layer is expected to help the cyclability of the electrodes by allowing a rapid transportation of Li ions^13^,^14^.

## Results and Discussion

**Figure 1:**
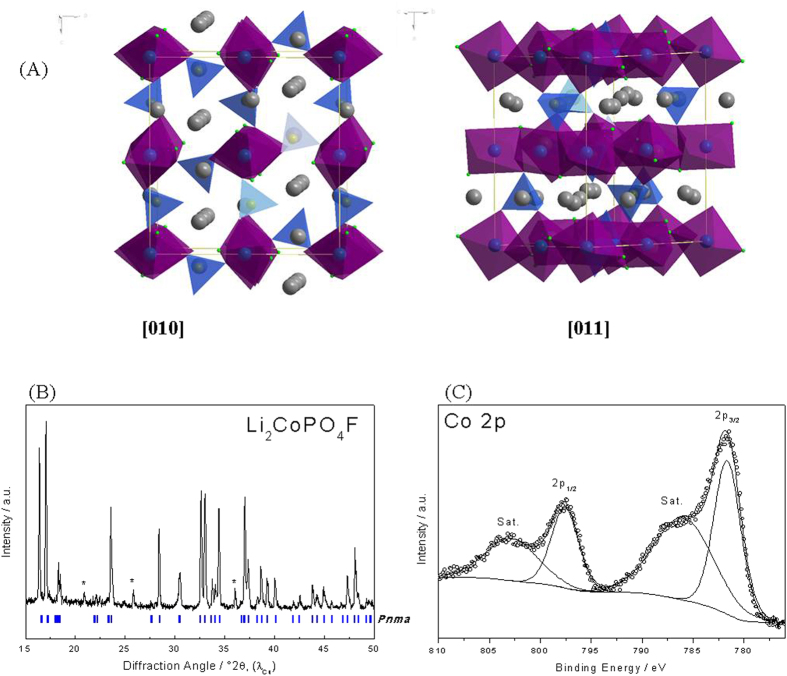
(**A**) Schematic view of the crystal structure of Li_2_CoPO_4_F along the [010] and [011] directions. (**B**) XRD pattern, and (**C**) XPS of the Co2p area from a Li_2_CoPO_4_F sample.

Figure 1A shows a schematic view of the Li_2_CoPO_4_F structure. It is formed by chains of CoO_4_F_2_ octahedra sharing their edges and interconnected with PO_4_ tetrahedral oxo-anions by corner sharing. The solid possesses an orthorhombic unit cell with *Pnma* space group, where there are 3 types of Li; Li1 in 8d sites and Li2 and Li3 in two sets of 4c sites. The Co is in 4a and 4b sites, the P in two sets of 4c sites, the F in two sets of 4c sites, and the O in four sets of 4c and two sets of 8d sites^6^. The cross linked structure forms an opened 3D framework, permitting Li ions to be inserted and extracted from multiple directions^15^,^16^,^17^. The XRD pattern of the synthesized Li_2_CoPO_4_F sample shown in Fig. 1B shows diffraction peaks indexed in agreement with the literature values, with a = 10.452 Å, b = 6.3911 Å, and c = 10.874 Å^6^,^17^. A LiCoPO_4_ impurity phase was detected (marked with symbol * in Fig. 1B), which could have been due to the relatively low heat treatment temperature^11^,^15^. The XPS signal of the Co2p, split into the 2p_3/2_ and 2p_1/2_ multiplet separated by 15.7 eV, is formed by a double peak at 781.7 eV and 786.2 eV (Fig. 1C), assigned to the Co^2+^in Li_2_CoPO_4_F in very good agreement with the observation reported in ref. 15.

**Figure 2:**
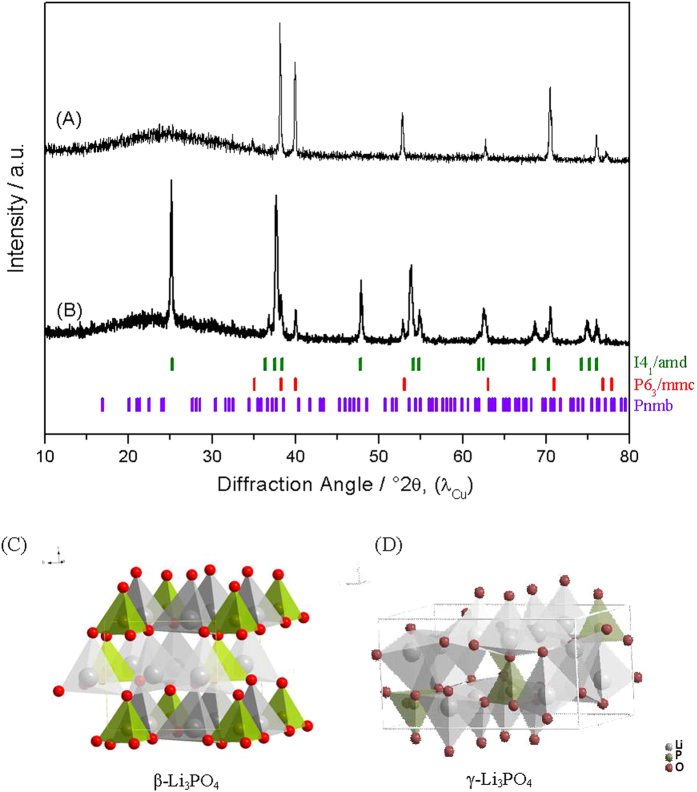
XRD patterns of Li_3_PO_4_-coated TiO_2_ anode (**A**) as-prepared and (**B**) after thermal annealing at 500 °C in air for 2 h. (**C,D**) show representations of the crystal structure of β-Li_3_PO_4_ and γ-Li_3_PO_4_, respectively.

The Li_3_PO_4_-coated TiO_2_ anode was prepared at room temperature, with some material also annealed at 500 °C in air for 2 h. The XRD patterns of the materials exhibit different features (Fig. 2). While for the as-prepared specimen, only Ti reflections (JCPDS file 05–0682 and space group *P6*_*3*_*/mmc*) can be observed, the annealed sample shows very intense peaks of anatase (JCPDS 21-1272 and space group *I4*_*1*_*/amd*). In both cases the presence of Li_3_PO_4_ could not be detected by XRD, due to the low crystallinity and small amounts of solid controlled by the conditions of the electrolytic deposition. Since the Li_3_PO_4_ is on the surface of TiO_2_ nanotubes further experiments allowed us to detected the (110), (101), (210) and (002) reflections of β-Li_3_PO_4_ (JCPDS 25–1030) phase for large time (20 min) and high current density (75 mA cm^−2^) during electrolytic deposition^13^. Previous experiments conducted to synthesize thick layers of lithium phosphate covering the complete surface of titania nanotubes^13^. So, this over 20 μm thick layer would passivate the active electrode and cannot be beneficial for such cycling purposes as electrodes in batteries. For this reason, we scaled down the fabrication of Li_3_PO_4_ layer to the nanometric size, observing that the best ratio of Li_3_PO_4_ to TiO_2_ is 9.03·10^−3^ g Li_3_PO_4_/g TiO_2_. By using optimal current densities and deposition times of ca. – 3.75 mA cm^−2^ and 1 min a finely dispersed layer of Li_3_PO_4_ can fill the titania nanotubes (see Supplementary Fig. S1 online). Under these conditions no diffraction peaks either of β-Li_3_PO_4_ or γ-Li_3_PO_4_ were detected in the X-ray diffraction patterns which were recorded from 10–80° (°2θ) with 0.02° of step size each 2 seconds as discussed above. Having a detailed inspection with HRTEM and SAED, the formation of β-Li_3_PO_4_ (at room temperature) or γ-Li_3_PO_4_ (when annealing) on titania nanotubes was unveiled as discussed below.

The lithium phosphate seems to play an important role enhancing the electrochemical response, both in Li half cells and in full Li-ion batteries, and deserves to be studied in more detail^13^,^17^,^18^,^19^. The β-Li_3_PO_4_ has a basic wurtzite structure where one position of the tetrahedral sites, T+ or T-, is fully occupied, along with cation ordering (Fig. 2C). It has twice the value of the unit cell along the axis when the phase transition from β- to γ-Li_3_PO_4_ (Fig. 2D) occurs. The γ-phase also consists of hexagonal close-packed oxide layers, but these are more distorted in comparison with the β-structure. Moreover, the cations are distributed over both sets of T+ and T- sites, leading LiO_4_ tetrahedra to share some of their edges, while only corner-sharing is present in the β-structure^20^,^21^,^22^.

**Figure 3:**
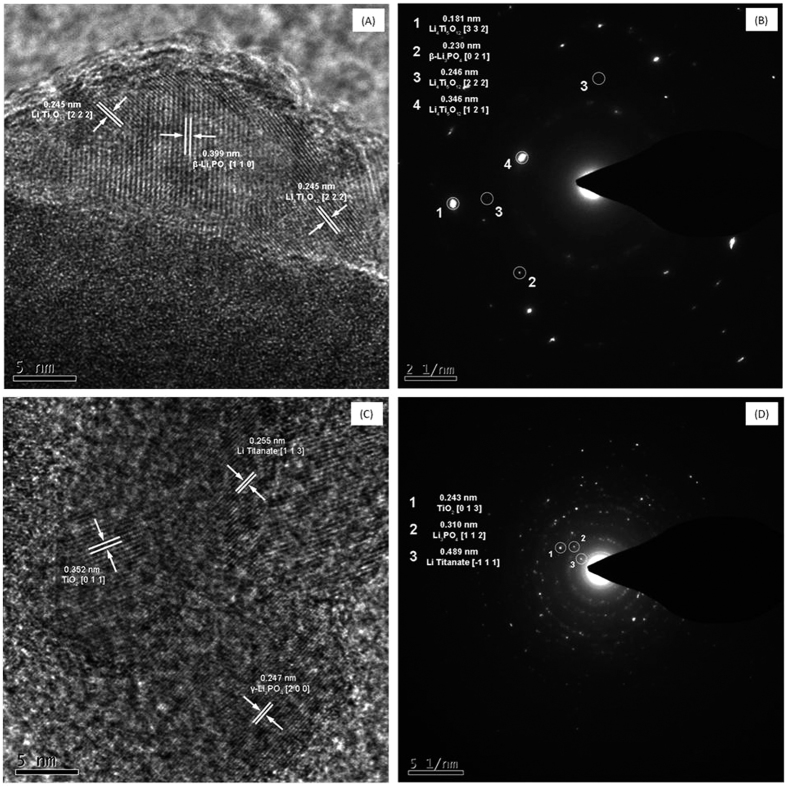
HRTEM and SAED imagery of (**A,B**) AD and (**C,D**) ADC samples, respectively.

In order to examine the formation of phosphate phases on titania nanotubes and discover whether or not an additional phase is formed at the interface, HRTEM and SAED measurements were performed. The HRTEM image of the “AD” sample (which was not calcined) is shown in Fig. 3A, which shows areas having visible lattice fringes measured and labelled according to their particular crystal structures. It can be seen that β-Li_3_PO_4_ was successfully formed, with the image containing a small region of ordered crystalline structure with a (110) interplanar spacing of 0.399 nm^23^. Here, amorphous TiO_2_ was not detected, but fringes corresponding to the (222) reflection of Li_4_Ti_5_O_12_ with an interplanar spacing of 0.245 nm were found^24^. This image was taken at the tip of the nanotube and we can observe that lithium titanate appeared at both sides of the region of lithium phosphate, which could explain the lack of TiO_2_ detection. The SAED data in Fig. 3B matches these d-spacings and confirms the presence of these materials. Figure 3C shows HRTEM imagery of the ADC sample (which was calcined), with regions of visible lattice fringes measured and labeled to identify their respective crystal structures. Here, a small region showing an ordered crystalline structure with a (011) interplanar spacing of 0.352 nm was detected, corresponding to anatase TiO_2_^25^. However, the observed form of lithium phosphate was γ-Li_3_PO_4_ as deduced from the (200) reflection (d_200_ = 0.247 nm), as expected after thermal annealing at 500 °C^26^. In addition, fringes corresponding to lithium titanate are also visible (d_113_ = 0.255 nm)^24^. The anatase TiO_2_, γ-Li_3_PO_4_ and Li_4_Ti_5_O_12_ regions are labeled “1”, “2” and “3” respectively to avoid confusion. The SAED data in Fig. 3D shows fine diffraction points corresponding to these three phases. The presence of small amounts of the Li_4_Ti_5_O_12_ phase at the interface between Li_3_PO_4_ and TiO_2_ gives an additional understanding of the structure of these composites.

The cathode half-cell reaction can be written as:


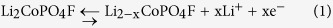


The theoretical capacity of Li_2_CoPO_4_F is 287 mA h g^−1^ for x = 2. However, recent studies have indicated that Li_2_CoPO_4_F can reach a maximum reversible capacity of 150 mA h g^−1^, with an outstanding high-voltage operation of ~5 V vs. Li^+ ^/Li^11^,^15^,^27^,^28^,^29^,^30^. Because of the high inefficiency from the first to the second cycle observable in Li_2_CoPO_4_F, these electrodes were subjected to activation cycles before being used in the complete lithium-ion cell (see Supplementary Fig. S2 online). The first-cycle irreversible capacity due to electrolyte decomposition was then avoided in the full cells^11^,^15^. Anatase is well known in the literature to exhibit a high reversibility in the first cycle and to operate at a safe average potential of 1.8–1.9 V vs. Li^+^/Li. The theoretical capacity delivered by anatase is around 167 mA h g^−1^ according to the following reaction:


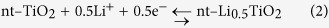


Taking into account their individual voltages, the combination of nt-TiO_2_ with Li_2_CoPO_4_F could give rise to a battery operating in the 3.1–3.2 V range. While considering the expected capacity of each electrode, the main overall reaction that may take place in the full cells can be summarized as follows:


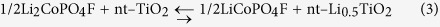


**Figure 4:**
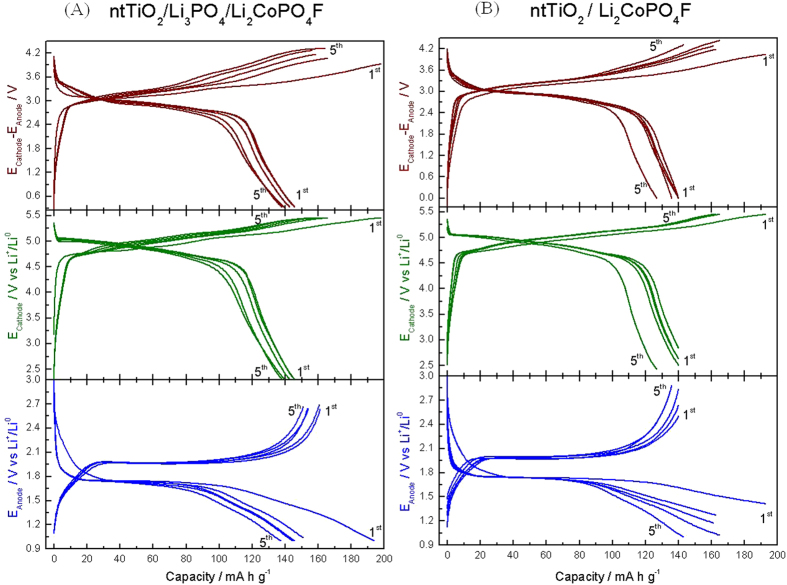
Galvanostatic charge/discharge cycles representing the E_Cathode_ – E_Anode_ vs. capacity of (**A**) nt-TiO_2_/γ-Li_3_PO_4_/Li_2_CoPO_4_F, and (**B**) nt-TiO_2_/Li_2_CoPO_4_F cells. This figure includes the voltage profiles of the cathode (E_Cathode_ vs. capacity) and the anode (E_Anode_ vs. capacity) in the middle and bottom plots. The capacity of the full cell is calculated using the cathode mass.

Capacity balance was carried out by assuming 140 mA h g^−1^ reversible capacity of the cathode after activation and 160 mA h g^−1^ of the anode (see Fig. 4), the resulting cathode mass to anode mass was: m+/m- = 1.14.

Figure 4A,B compare the reversible voltage profile versus Li of the TiO_2_ with and without Li_3_PO_4_ (bottom) and of the Li_2_CoPO_4_F cathode (middle). The anode operates reversibly with continuous, plateaued charge-discharge curves with a reversible capacity of 150 mA h g^−1^ at an average voltage value of about 1.8–1.9 V, while the Li_2_CoPO_4_F cathode cycles with a reversible capacity of 148 mA h g^−1^ at a voltage value of 5 V vs. Li with a flat plateau, typical of the two phase reaction of lithium-cobalt fluorophosphates^19^.

The upper plots of Fig. 4A,B show the trend of the full-cell voltage profile, demonstrating very stable behaviour. The cell operates with an average voltage of around 3.1–3.2 V, while the voltage profile is the combination of the flat voltage of the Li_2_CoPO_4_F cathode (Fig. 4 middle plots) and the flat voltage of the TiO_2_ (Fig. 4B bottom plot) or TiO_2_/γ-Li_3_PO_4_ anodes (Fig. 4A bottom plot). The reversible capacity of the full battery measured at a state of discharge is about 150 mA h g^−1^, reaching about 99% of the maximum reversible capacity. The achieved energy density is 472 W h kg^−1^, an enhanced value as compared to the majority of published batteries^31^,^32^,^33^,^34^,^35^,^36^.

Li_4_Ti_5_O_12_ is a well-known material for LIBs and typically shows a stable plateau at 1.54 V (vs. Li^+^/Li)^7^,^37^,^38^,^39^. However, such a plateau is not visible in the charge/discharge curves (Fig. 4A,B bottom plots). Instead, the typical plateau of anatase TiO_2_ can be seen. The lithium titanate phase is formed in a minute fraction at the interphase between TiO_2_ and Li_3_PO_4_, as detected by SAED and HRTEM measurements (Fig. 3). However, this phase was not detected by XRD (Fig. 2), due to its particularly low proportion. Then, its contribution to battery functionality is expected to be negligible.

**Figure 5:**
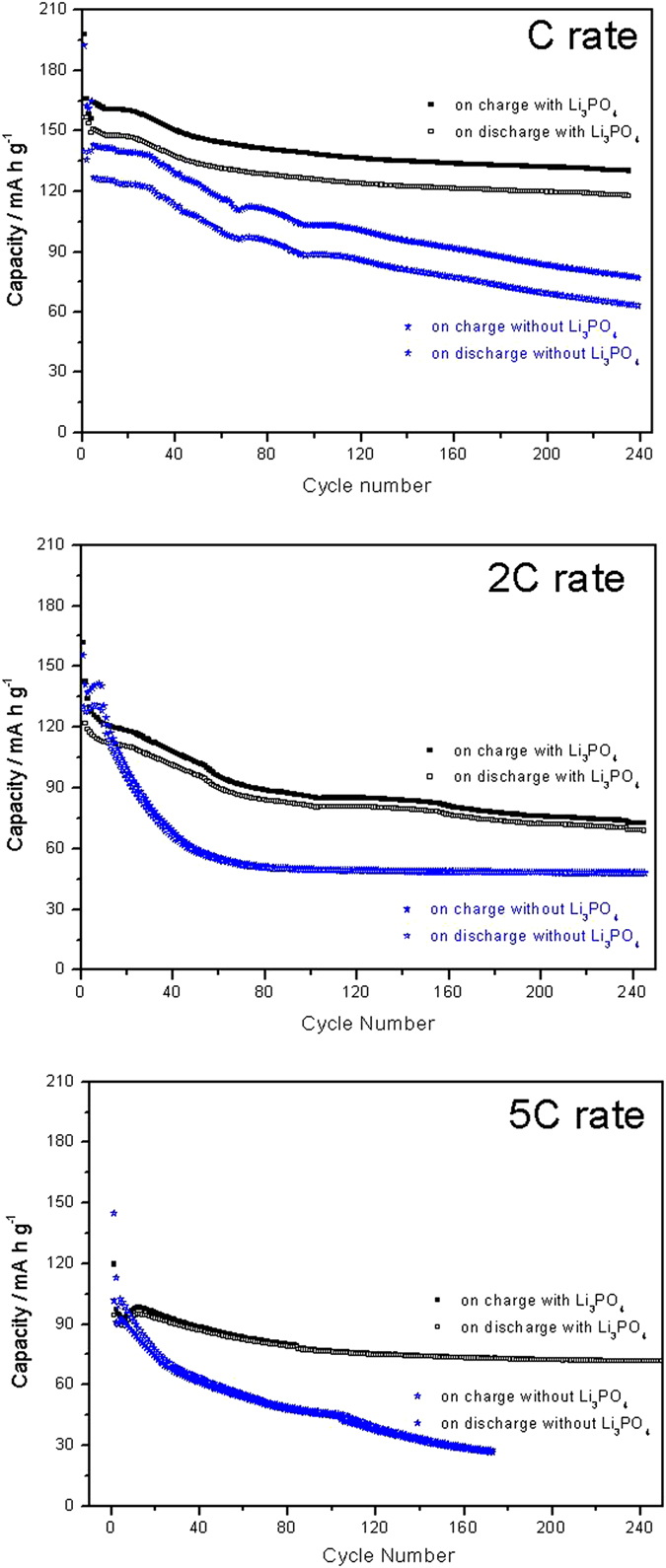
A comparison of specific capacity versus cycle number between nt-TiO_2_/Li_2_CoPO_4_F, and nt-TiO_2_/γ-Li_3_PO_4_/Li_2_CoPO_4_F cells, calculated at 1 C, 2 C, and 5 C rates.

The stability of chosen electrode materials is another key factor for battery cycling. Figure 5 compares the cycling stability of TiO_2_/Li_3_PO_4_/Li_2_CoPO_4_F and TiO_2_/Li_2_CoPO_4_F measured at 1 C, 2 C and 5 C rates. The battery that utilizes Li_3_PO_4_ shows very good cycling behaviour as compared to that of the full cell without Li_3_PO_4_, operating at 1 C, 2 C and 5 C rates for more than 240 charge-discharge cycles with high coulombic efficiencies of 79, 62 and 73%, respectively. These differences were found significant from a statistical analysis of cycling experiments of five different cells for each composition that can be found as Supplementary Table S1 online. As expected, capacity decay from 150 mA h g^−1^ at 1 C to 120 mA h g^−1^ is recorded at 2 C, and to 90 mA h g^−1^ at 5 C. This battery, based on TiO_2_/Li_3_PO_4_/Li_2_CoPO_4_F, exhibits much better performance in terms of cyclability and coulombic efficiency than TiO_2_/Li_2_CoPO_4_F and that previously reported and based on Li_4_Ti_5_O_12_ as an anode material^11^. The excellent performance of this battery observed in terms of specific capacity, cycling life and rate capability is to the best of our knowledge only seldom reported, and confirms the great potential of TiO_2_/Li_3_PO_4_ as an innovative electrode material that can aid the progress of lithium-based energy storage systems.

## Conclusions

The high working voltage and excellent rate capability observed of the TiO_2_/Li_3_PO_4_/Li_2_CoPO_4_F full cell makes it a promising high energy density LIB with acceptable rate performance (472 W h kg^−1^ at a 1 C rate, and 284 W h kg^−1^ at a 5 C rate), preventing the emergence of safety issues caused by the highly reactive lithiated graphite present in most LIB systems. The existence of Li_3_PO_4_ and the minute fraction of Li_4_Ti_5_O_12_ present between the TiO_2_/ Li_3_PO_4_ interfaces can explain the good cyclability of the full cell as this inactive matrix allows rapid transportation of the lithium ions.

## Methods

The Li_2_CoPO_4_F/C nanocomposite was synthesized by the sol-gel (SG) method as previously reported^11^,^15^. The self-organized titania nanotube (nt–TiO_2_) layer was fabricated by an anodization process using Ti foils at 60 V for 2 h, with a freshly prepared mixture of EG/water (92:8 vol.) containing 0.3 wt. % NH_4_F as an electrolyte solution. The deposition of electrolytic Li_3_PO_4_ was performed on nt-TiO_2_ as either amorphous material (labeled as AD) or, after calcination, as a anatase material (labeled as ADC), using a current density of -3.75 mA cm^−2^ for 1 min. Electrolytic Li_3_PO_4_ films were deposited by an electrochemical procedure consisting of proton reduction with a subsequent local increase of pH in the vicinity of the substrate surface, hydrogen phosphate dissociation and Li_3_PO_4_ deposition on the surface of the cathode^13^. Optional thermal annealing at 500 °C was performed. The thickness and active mass of the anode was 8 μm and 0.935 mg cm^−2^ respectively^13^.

HRTEM and SAED images were collected with a Tecnai F-20 device operating at 200 kV. The X-ray diffraction (XRD) patterns were recorded with a Siemens D5000 instrument utilizing Cu Kα radiation. The X-ray photoelectron spectroscopy (XPS) measurements were performed with a SPECS Phobios 150MCD instrument using a Mg Kα source (1253.6 eV) and a chamber pressure of 4 × 10^**−**9^ mbar.

Electrochemical characterization and cycling properties (discharge**−**charge) were performed using a three electrode configuration with a Biologic-VMP instrument. The full cells were assembled in a glovebox under an Ar atmosphere. A 9 mm diameter lithium disk was used as reference electrode, with Li_3_PO_4_**−**ntTiO_2_-based films and Li_2_CoPO_4_F used as counter and working electrodes. The electrolyte solution was 1 M LiPF_6_ (EC:DEC) embedded in Whatman glass fiber disks. The full cell was cycled at 1C, 2 C and 5 C rates (C = 0.3 mA cm^−2^). The activation of the positive electrode (Li_2_CoPO_4_F) offers the possibility of achieving a remarkable reversible capacity for the full cell. In the present study, the activation step of the Li_2_CoPO_4_F consisted of two successive cycles of galvanostatic charging to 5.4 V, followed by discharging to 3.0 V, at a 100 mA g^-1^ current density and using metallic Li as a counter electrode. When designing the full battery, it is quite important to obtain an optimal balance of cathode and anode both in terms of weight and electrochemical properties. The calculation of the energy density of the battery only considered the specific capacity and the working potential (E_cathode_ – E_anode_) of the full battery, without further consideration of the mass of the active materials, electrolyte and packing materials.

## Additional Information

**How to cite this article**: Ortiz, G. F. *et al.* Enhancing the energy density of safer Li-ion batteries by combining high-voltage lithium cobalt fluorophosphate cathodes and nanostructured titania anodes. *Sci. Rep.* **6**, 20656; doi: 10.1038/srep20656 (2016).

## Supplementary Material

Supplementary Information
