# Supplementary material for: Enhancing the energy density of safer Li-ion batteries by combining high-voltage lithium cobalt fluorophosphate cathodes and nanostructured titania anodes
Source: Sci Rep. 2016 Feb 16;6:20656. doi: 10.1038/srep20656 (PMC4808834; doi:10.1038/srep20656)
Supplement: Supplementary Information [file srep20656-s1.doc]

Supplementary Information

**Enhancing the energy density of safer Li-ion batteries by combining high-voltage lithium cobalt fluorophosphate cathodes and nanostructured titania anodes**

Gregorio F. Ortiz ^a^,*, María C. López ^a^, Yixiao Li ^b^, Matthew J. McDonald ^b^, Marta Cabello^a^, José L. Tirado ^a^, and Yong Yang ^b^

^a^ *Inorganic Chemistry Laboratory, University of Córdoba, Marie Curie Building, Campus of Rabanales, E-14071 Córdoba, Spain,* *Corresponding author, email [q72maorg@uco.es](mailto:q72maorg@uco.es) ,Tel.: 0034-957-218637

^b^ *State Key Laboratory of Physical Chemistry of Solid Surfaces, Department of Chemistry, College of Chemistry and Chemical Engineering, Xiamen University, Xiamen 361005, P. R. China, email:* [yyang@xmu.edu.cn](mailto:yyang@xmu.edu.cn) , Tel.: 0086-592-2185753

**
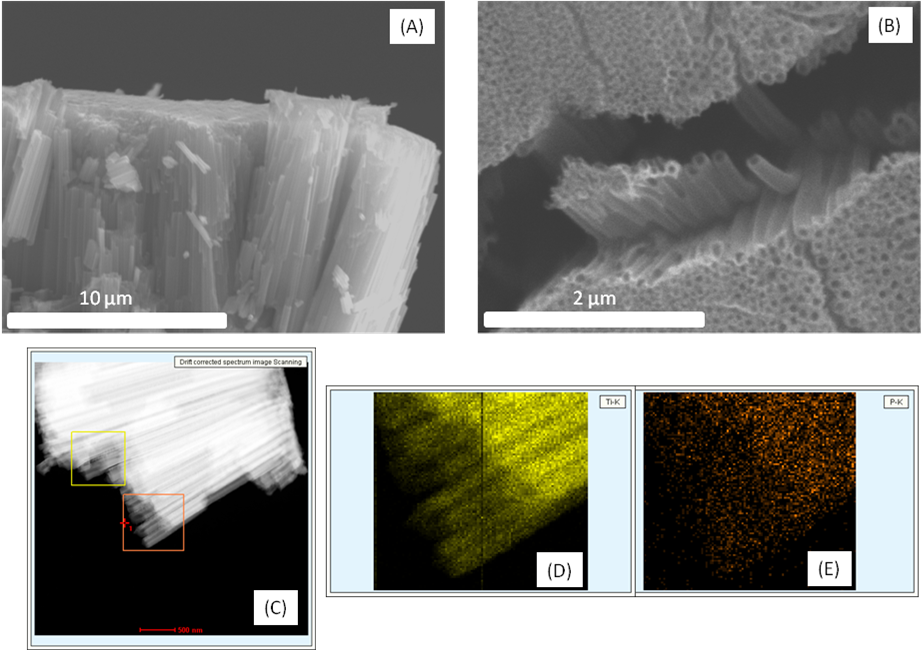
**

**Figure S1**. (A) Cross sectional and (B) top view SEM images of deposited Li_3_PO_4_ on titania nanotubes after annealing at 500 ºC. (C) The corresponding dark-field TEM image and (D) Ti-K and (E) P-K EDAX analyses from previous TEM image.

**Figure S2**. The first charge/discharge profile of the Li_2_CoPO_4_F sample at 1C rate using a liquid electrolyte containing 1 M LiPF_6_ (EC:DMC).

**Table S1.** Statistical analysis of the capacity on discharge obtained for the nt­TiO_2_/ γ­Li_3_PO_4_/ Li_2_CoPO_4_F and nt­TiO_2_/ Li_2_CoPO_4_F cells, cycled at 1 C.

| Treatment | Cathode mass (mg) | 1^st^ Reversible Capacity  (mA h g^-1^) | 120^th^ Reversible Capacity  (mA h g^-1^) |
| --- | --- | --- | --- |
| With Li_3_PO_4_ | 1.30 | 156.7 | 124.4 |
|  | 1.17 | 154.0 | 123.7 |
|  | 1.19 | 151.8 | 119.6 |
|  | 1.15 | 152.5 | 122.4 |
|  | 1.05 | 149.3 | 121.0 |
| Mean±s |  | 152.9±2.7 | 122.2±2.0 |
| Without Li_3_PO_4_ | 1.30 | 141.7 | 85.4 |
|  | 1.19 | 139.8 | 85.8 |
|  | 1.15 | 140.2 | 81.4 |
|  | 1.1 | 137.6 | 83.0 |
|  | 1.05 | 134.3 | 78.7 |
| Mean±s |  | 138.7±2.9 | 82.9±2.9 |
